# Robotic Splenectomy as a Salvage Therapy Post Failed Splenic Embolization in Chronic Immune Thrombocytopenic Purpura Due to the COVID-19 Vaccine

**DOI:** 10.7759/cureus.81536

**Published:** 2025-03-31

**Authors:** Danielle E Canfield, Alejandro Pizano, Tina Joseph

**Affiliations:** 1 General Surgery, Nassau University Medical Center, East Meadow, USA

**Keywords:** covid-19 vaccine complications, immune thrombocytopenic purpura (itp), robotic splenectomy, splenic artery embolization, thrombocytopenia

## Abstract

Refractory immune thrombocytopenic purpura (ITP) is a rare autoimmune condition that does not respond to medical treatment and poses significant challenges in management due to the risk of severe bleeding. This report discusses the challenges in managing a 65-year-old male patient with ITP secondary to the second COVID-19 vaccine and refractory to medical and surgical therapy who underwent robotic splenectomy. After failing multiple cycles of high-dose corticosteroids, IVIG (intravenous immunoglobulin), and romiplostim, the patient underwent sub-selective splenic artery embolization (SAE). After 26 months, the spleen retained its size, and he underwent a second sub-selective SAE followed by an elective robotic splenectomy, which converted to an open procedure due to intraoperative bleeding. Seven days post-op, the patient's platelets rebounded to adequate levels, and he was discharged on post-op day eight. The patient had evidence of thrombocytopenia at follow-up on postoperative day 24 that rebounded by postoperative day 66. This patient's unique treatment course highlights various medical and surgical challenges in the armamentarium for patients with ITP.

## Introduction

Immune thrombocytopenic purpura (ITP) is an autoimmune condition caused by IgG autoantibodies that bind to and help facilitate the destruction of platelets, leading to a platelet count of less than 100 K/mm^3^ and/or bleeding episodes. This is typically a diagnosis of exclusion and can be due to other autoimmune conditions, drugs, viral illnesses, or vaccines [1]. The COVID-19 vaccines from both Pfizer and Moderna have been documented to cause thrombocytopenia, although it is a very rare complication at a rate of 0.80 per million doses [2]. The mechanism of ITP after a vaccine is unknown but thought to be due to either an immune reaction mounted in response to the vaccine or preformed antibodies against components of the vaccine [3]. When unresponsive to the first-line medications, such as corticosteroids and intravenous immunoglobulins (IVIG), ITP can be deemed chronic, and those cases unresponsive to splenectomy are deemed to be refractory, which poses significant challenges in management due to the risk of severe bleeding [1]. This case report was previously presented as a poster at the 2024 NuHealth Academic Research Day on May 17, 2024.

## Case presentation

A 65-year-old male patient with no significant past medical history presented six days after his second dose of the Moderna COVID-19 vaccine, complaining of diffuse purpura on all extremities, two episodes of melena, dizziness, and fatigue. Initial laboratory studies in the emergency department revealed a platelet count of less than 10,000 K/mm^3^. The initial workup included a complete blood count, comprehensive metabolic panel, coagulation panel, blood smear, viral panel, gastroenterology consult, hematology consult, and a complete history and medication list. After differential diagnoses such as heparin-induced thrombocytopenia, thrombotic thrombocytopenic purpura, disseminated intravascular coagulation, viral infection, and hematological malignancies were ruled out, the patient was diagnosed with ITP. He was initially managed with medical therapy consisting of corticosteroids, IVIG, and eventually the addition of romiplostim, a thrombopoietin (TPO) activating agent, on an inpatient and outpatient basis with no sustained improvement of platelets.

Four months after the initial presentation, the patient was found to have splenomegaly measuring 15.9 x 4.2 cm and was deemed to be a candidate for surgical management. After denying a total splenectomy, the patient agreed to a sub-selective splenic artery embolization (SAE) with partial retention of splenic function. The patient tolerated the procedure well, after which he was continued on medical management with no sustained rebound in platelets.

**Figure 1:**
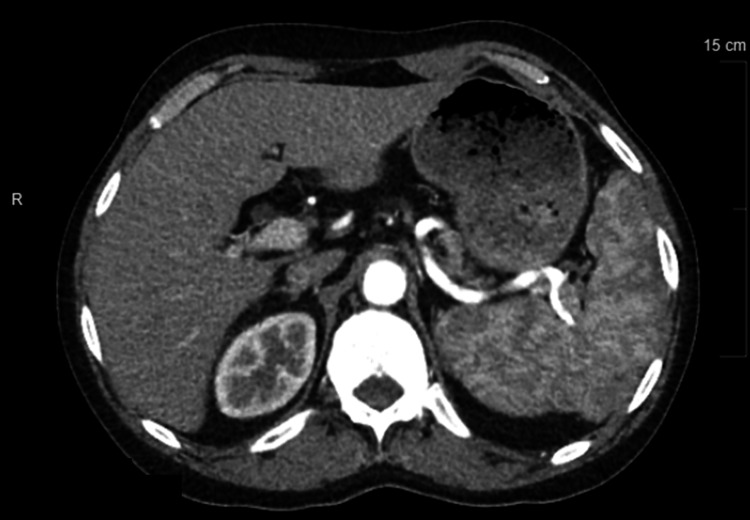
Preoperative axial CT with contrast showing heterogeneous uptake in the enlarged spleen.

Twenty-six months after the first SAE, the patient was still having thrombocytopenia with multiple bleeding episodes and was found to have persistent splenomegaly measuring 14.8 x 5.8 x 6.7 cm. Figure 1 depicts the patient's CT scan.

**Figure 2:**
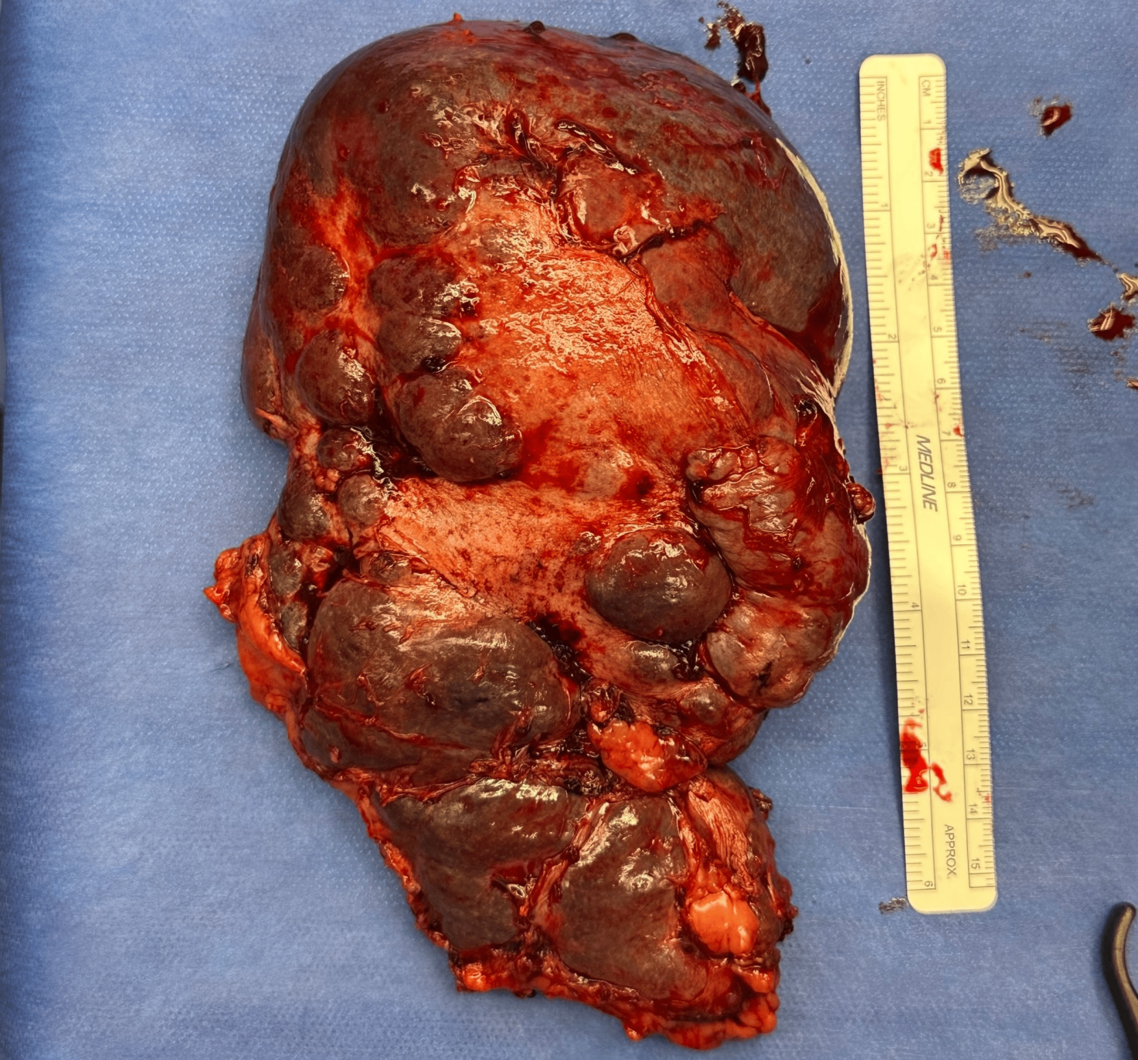
The patient's spleen post resection with areas of necrosis, measuring greater than 15 cm and weighing 450 g.

Definitive surgical management was discussed with the patient, to which he was hesitant. He agreed to undergo a second sub-selective SAE. When this did not produce a meaningful increase in platelets, he was agreeable to a robotic splenectomy 15 days after the SAE. The patient was optimized with IVIG and transfusion of two units of platelets to decrease bleeding risk before the operation. During the robotic procedure, there was bleeding noted from a possible splenic laceration vs. collateral splenic vessels, and the decision was made to convert the procedure to an open splenectomy. Hemostasis was achieved via ligation of collateral splenic vessels, and the spleen was successfully removed. Bleeding was thought to be an unfortunate complication due to the patient's complex disease course and unwillingness to undergo definitive surgical management earlier in his disease course. The patient was extubated and brought to the post-anesthesia care unit in stable condition. The gross pathology of the patient's spleen can be seen below (Figure 2).

His immediate postoperative course was complicated by intra-abdominal bleeding due to persistent severe thrombocytopenia despite temporizing platelet transfusions. The patient promptly returned to the operating room, where significant oozing was noted under the left diaphragm. Manual pressure was held, hemostatic agents were applied, and a small collateral vessel was ligated, resulting in adequate hemostasis. No further bleeding was noted, and the left upper quadrant was packed with Nu-Knit and Surgicel powder (Ethicon, Raritan, New Jersey). The patient returned to the surgical ICU and remained in stable condition. On postoperative day two, the patient complained of sudden-onset shortness of breath, and a chest X-ray showed pulmonary edema. The patient was diagnosed with transfusion-associated circulatory overload due to the large burden of blood products he was given over the previous 48 hours. He was treated with diuretics, leading to a full recovery of symptoms and respiratory function.

Seven days post-op, the patient's platelets rebounded to 302 K/mm^3^ with no other complications. He was discharged from the surgical service and transferred to the inpatient rehabilitation floor for decreased mobility on post-op day eight. On postoperative day 10, the patient's platelets were noted to be 571 K/mm^3^. Despite current guidelines, the patient was started on 81 mg of aspirin daily by the hematology team due to the rapid increase in his platelet numbers. The patient was discharged home with regular outpatient follow-up scheduled on postoperative day 11.

**Figure 3:**
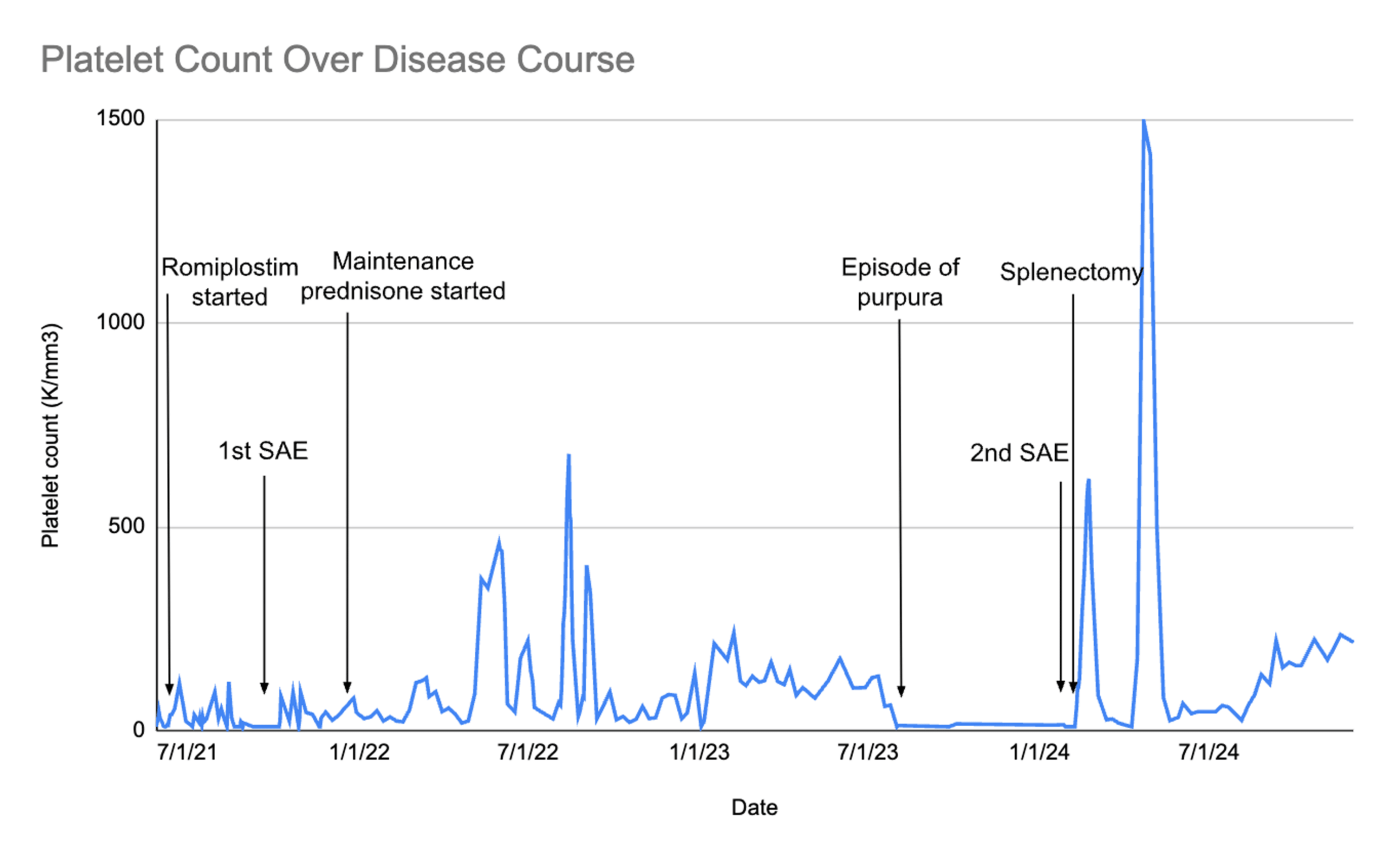
Graph depicting the patient's platelet count over the total course of his disease with notable moments in treatment. SAE: splenic artery embolization

On postoperative day 24, the patient was seen in an outpatient hematology clinic and found to have platelets of 86 K/mm^3^; aspirin was stopped, and reevaluation on post-op day 53 showed platelets of 14 K/mm^3^. The patient had no bleeding episodes during this time. The patient underwent a nuclear medicine scan, which ruled out the presence of accessory splenic tissue. He was continued on medical management of corticosteroids, IVIG, and romiplostim with a rebound in platelets to 177 K/mm^3^ on postoperative day 63. Platelet levels then spiked to 1493 K/mm^3^ on postoperative day 73 and returned to 25 K/mm^3^ on postoperative day 101. Platelet levels were found to be approximately 100 K/mm^3^ at the six-month follow-up, and they remained stable at 200 K/mm^3^ at the 10-month follow-up. Figure 3 presents a comprehensive overview of the patient's platelet course.

The patient continued to follow up weekly with the hematology team for platelet level checks. He has been able to return to his lifestyle pre-ITP diagnosis and has had no bleeding episodes since the splenectomy.

## Discussion

Managing chronic ITP presents considerable challenges, particularly in cases refractory to conventional treatments. This case report underscores the complexities encountered in treating a 65-year-old male patient with ITP secondary to the second COVID-19 vaccine, which has been documented as one of the new vaccine-induced ITPs.

First-line therapy for ITP consists of medical management with corticosteroids and IVIG. These pharmacotherapies work together to increase platelet counts and rapidly resolve bleeding episodes; however, they often only have transient effects [4]. Second-line medical therapy consists of thrombopoietin-receptor agonists and rituximab, an anti-CD20 monoclonal antibody, and often achieves a longer-lasting period of remission [4]. Patients who are given a TPO agonist such as romiplostim typically see a platelet response starting approximately two weeks after beginning treatment [5]. Patients who fail medical treatment can be managed operatively. Most of the COVID-19 or vaccine-induced ITPs have positive responses to medical management [6]. However, our case presented as refractory to extensive trials of medical and intervention therapies, including sub-selective SAE, following the American Society of Hematology guidelines for ITP treatment [7].

In an observational study of 91 patients with steroid-resistant ITP, a partial splenic embolization was an effective treatment in 84% of patients, making this an adequate alternative treatment modality for patients with chronic ITP who decline the more invasive total splenectomy or have other cardiovascular risk factors [8]. After failing both medical therapy and conservative surgical management, our patient underwent a second sub-selective SAE in an attempt to reduce the size of the spleen prior to robotic splenectomy and minimize bleeding during the procedure.

A splenectomy has become the mainstay of treatment for patients who fail multiple rounds of medical therapy after 12 months. This procedure removes the main site of platelet destruction and antibody formation, thereby leading to long-term remission in patients with ITP [9]. It is measured that around 70% of patients will achieve a complete, long-term platelet response post-splenectomy [10].

Postoperatively, our patient has had severe fluctuations in his platelet levels, not typically seen after definitive management with splenectomy. One potential explanation for this patient's lack of sustained response to splenectomy is connected to his prior treatment with romiplostim. Two months prior to his splenectomy, the patient's bone marrow biopsy showed morphologic findings strongly suggestive of myelofibrosis with markedly increased reticulin stain and increased collagen on trichrome stain. Although a rare complication, romiplostim and other TPO-activating agents have been shown to cause bone marrow fibrosis, especially when used at the maximum dosage, due to increased production of reticulin fibers within the bone marrow [11]. This patient received both romiplostim and eltrombopag at maximum dosages for a lengthy period of time prior to his splenectomy and a few doses after the splenectomy. This myelofibrosis can affect the multipotent stem cells of the bone marrow and be a potential cause of his prolonged thrombocytopenia.

This case underscores the complexities of managing severe refractory ITP post-COVID-19 vaccination, highlighting the potential complications of advanced medical and surgical therapies and the necessity for personalized treatment strategies aligned with a multidisciplinary team.

## Conclusions

This patient's unique treatment course highlights various challenges in the treatment of patients with vaccine-induced chronic ITP, illustrating the challenges faced when conventional medical treatments, interventional procedures, and surgical interventions fail. The patient's journey from high-dose corticosteroids, IVIG, and romiplostim to repeated sub-selective SAE and ultimately a complicated robotic splenectomy was longer and more complicated than most cases but did eventually lead to a stabilization of platelets at a therapeutic level. The postoperative fluctuations in platelet levels emphasize the unpredictability of treatment outcomes and the necessity for ongoing research and innovation in therapeutic strategies for ITP. This case also adds to the continually evolving research on the repercussions of the COVID-19 vaccine and different mainstays of management. Each patient with ITP will have a unique disease course and treatment response, which underscores the need for a tailored approach in refractory ITP cases to achieve long-term platelet stability.
